# Activation of Chymotrypsin-Like Activity of the Proteasome during Ischemia Induces Myocardial Dysfunction and Death

**DOI:** 10.1371/journal.pone.0161068

**Published:** 2016-08-16

**Authors:** Gina Sanchez, Daniela Berrios, Ivonne Olmedo, Javier Pezoa, Jaime A. Riquelme, Luis Montecinos, Zully Pedrozo, Paulina Donoso

**Affiliations:** 1 Programa de Fisiopatología, Instituto de Ciencias Biomédicas, Facultad de Medicina, Universidad de Chile, Santiago, Chile; 2 Centro de Estudios Moleculares de la Célula, Facultad de Medicina, Universidad de Chile, Santiago, Chile; 3 Programa de Fisiología y Biofísica, Instituto de Ciencias Biomédicas, Facultad de Medicina, Universidad de Chile, Santiago, Chile; 4 Advanced Center for Chronic Diseases, Facultad de Ciencias Químicas y Farmacéuticas, Universidad de Chile, Santiago, Chile; Indiana University School of Medicine, UNITED STATES

## Abstract

Inhibitors of the ubiquitin-proteasome system improve hemodynamic parameters and decrease the infarct size after ischemia reperfusion. The molecular basis of this protection is not fully understood since most available data report inhibition of the 26 proteasome after ischemia reperfusion. The decrease in cellular ATP levels during ischemia leads to the dissociation of the 26S proteasome into the 19S regulatory complex and the 20S catalytic core, which results in protein degradation independently of ubiquitination. There is scarce information on the activity of the 20S proteasome during cardiac ischemia. Accordingly, the aim of this work was to determine the effects of 30 minutes of ischemia, or 30 min of ischemia followed by 60 minutes of reperfusion on the three main peptidase activities of the 20S proteasome in Langendorff perfused rat hearts. We found that 30 min of ischemia produced a significant increase in the chymotrypsin-like activity of the proteasome, without changes in its caspase-like or trypsin-like activities. In contrast, all three activities were decreased upon reperfusion. Ixazomib, perfused before ischemia at a concentration that reduced the chymotrypsin-like activity to 50% of the control values, without affecting the other proteasomal activities, improved the hemodynamic parameters upon reperfusion and decreased the infarct size. Ixazomib also prevented the 50% reduction in RyR2 content observed after ischemia. The protection was lost, however, when simultaneous inhibition of chymotrypsin-like and caspase-like activities of the proteasome was achieved at higher concentration of ixazomib. Our results suggest that selective inhibition of chymotrypsin-like activity of the proteasome during ischemia preserves key proteins for cardiomyocyte function and exerts a positive impact on cardiac performance after reperfusion.

## Introduction

Myocardial ischemia represents a severe cellular stress that triggers dramatic biochemical and metabolic changes in the heart. The generation of reactive oxygen species (ROS) during ischemia [1–3] initiates the oxidation and modification of cellular proteins by lipid hydroperoxides [4] that eventually produce irreversible cell damage and death. The length of the ischemic period is a critical determinant of cell survival or death. Reperfusion is essential to keep cells alive, but the burst of ROS generation and calcium overload that takes place upon reperfusion further increases cellular damage [5,6].

Protein degradation during ischemia provides aminoacids to be used as substrates for mitochondrial energy production and avoids the accumulation of toxic aggregates. Proteasomes are proteolytic complexes responsible for the degradation of over 90% of cellular proteins. The 26S proteasome, composed by the 20S catalytic core plus the 19S regulatory complex, mediates the ATP-dependent degradation of ubiquitinated proteins while the 20S proteasome, that contains the catalytic subunits, degrades oxidized proteins independent of ubiquitination. Both, the 20S proteasome and the 26S proteasome coexist in the heart [7,8]. Inhibition of the proteasome as a pharmacological strategy to prevent cell damage in ischemia reperfusion has produced conflicting results. Several studies report that the activity of the 26S proteasome decreases after ischemia reperfusion [9–11] and that further pharmacological inhibition produces more damage [12]. Conversely, an increasing number of studies have shown that proteasome inhibitors protect the heart from IR damage [13–16].

The decrease in cellular ATP content that occurs during ischemia promotes the dissociation of the 20S catalytic core from its associated regulatory particles in the 26S proteasome [17]. There is scarce information on the activity of the 20S proteasome during ischemia. Accordingly, the aim of this work was to determine this activity in isolated rat hearts and to evaluate the effect of ixazomib, the first oral proteasome inhibitor approved by the FDA for the treatment of multiple myeloma, on ischemia reperfusion injury.

## Methods

Male Sprague-Dawley (SD) rats (220–240 g) were obtained from the animal facility of the School of Medicine, University of Chile. Rats were kept at a temperature of 22 ± 3°C and 12 h light-dark cycle, with free access to standard food and water. All procedures in this study conform to the Guide for the Care and Use of Laboratory Animals, published by the U.S. National Institutes of Health (NIH, Publication No. 85–23, revised in 1996), and were approved by the Institutional Ethics Committee of the School of Medicine, Universidad de Chile (Protocol CBA#0399FMUCH).

### Experimental protocol

SD male rats were anesthetized with pentobarbital (80 mg/kg, intraperitoneal) and heparin 100 U/kg was injected into the right atria. The heart was rapidly excised, mounted in a temperature regulated heart chamber and perfused at 37°C via the ascending aorta using a peristaltic infusion pump at a constant flow of 10–14 mL/min. The Krebs Henseleit solution contained (in mmol/L): 128.3 NaCl, 4.7 KCl, 1.35 CaCl_2_, 1.1 MgSO_4_, 20.2 NaHCO_3_, 0.4 NaH_2_PO_4_, pH 7.4, and 11.1 glucose, equilibrated with a gas mixture of 95% O_2_/5% CO_2_. Left ventricular hemodynamic parameters were measured with a latex balloon inserted into the left ventricle and connected to a pressure transducer. After 20 min of stabilization, the hearts were subjected to 30 min of global ischemia at 37°C. Hearts were either frozen in liquid N_2_ immediately after ischemia or perfused with Krebs Henseleit oxygenated solution for 60 min before freezing. The proteasome inhibitor ixazomib (MLN 9708, Selleckchem, Houston, TX, USA) was perfused during 10 min before ischemia at concentrations of 0.1 or 1μmol/L. To compare with published data, MG132 (Merck Millipore, Billerica, MA, USA) was perfused as above at concentrations of 0.5 or 6 μmol/L. Control hearts, not subjected to ischemia, were perfused with or without proteasome inhibitors for 50 (control for ischemia) or 110 min (control for ischemia–reperfusion). At the end of the reperfusion period, hearts were snap frozen in liquid N_2_ or perfused with triphenyl tetrazolium (TTC) to measure the infarct size.

### Preparation of whole ventricle homogenates

Frozen ventricles were reduced to powder under liquid N_2_ and homogenized under slightly different conditions according to the ensuing biochemical determination. To measure the activity of the proteasome, frozen tissue powder was homogenized in 5 volumes of a solution containing (in mmol/L) 50 NaCl, 1 Na_2_EDTA, 10 HEPES-NaOH, pH 8.0, 250 Sucrose, 0.2% Triton X-100, as described [18]. This fraction was prepared without reducing agents or protease inhibitors just before the determination of proteasome activity.

To measure [^3^H]-ryanodine binding, the frozen powder was homogenized in 4 volumes of a solution containing (in mmol/L) 300 sucrose, 20 MOPS-Tris buffer, pH 7.0, with protease inhibitors (1 mmol/L PMSF, 1 mmol/L benzamidine, 2 μg/mL leupeptine, 1 μg/mL pepstatin). Unbroken cells and debris were eliminated by centrifugation at 600 x g for 10 min, at 4°C. The supernatant (whole homogenate) was fractioned into small aliquots, frozen in liquid N_2_ and kept at -80°C. For western blot analysis, the frozen tissue was homogenized as above, but the buffer contained in addition 2 mmol/L EDTA, 2 mmol/L EGTA and the detergents NP-40 (1%) and SDS (1%). The supernatant was recovered by centrifugation at 1000 x g for 20 min at 4°C. Aliquots were frozen in liquid N_2_ and kept at -80°C as above. Protein concentration was determined by the method of Hartree [19].

### Proteasome activity

The three main peptidase activities of the proteasome were determined in the absence of reducing agents as described before [18]. Briefly, heart homogenates (0.3 mg protein/mL) were incubated with fluorogenic proteasome substrates in a solution containing (mmol/L) 50 NaCl, 1 EDTA, 250 sucrose, 10 HEPES-NaOH, pH 8.0. The substrates used were Suc-LLVY-amc (21 μmol/L) for chymotrypsin-like activity, Z-LLE-amc (105 μmol/L) for caspase-like activity and Boc-LSTR-amc (34 μmol/L) for trypsin-like activity. Fluorescence was measured at 30°C in a plate reader at 380 nm excitation / 440 nm emission wavelengths. Non-specific proteolysis was determined in the presence of MG132 (30 μmol/L). All proteasome substrates were obtained from Sigma-Aldrich (St Louis, MI).

### Western blots

Proteins were separated by electrophoresis in 3%–8% Tris-Acetate gels (Criterion XT, Bio-Rad, Hercules, CA) under reducing conditions. After transfer to polyvinylidene difluoride membranes, proteins were probed with anti-RyR2 antibody (Thermo Scientific; Rockford, IL) or anti-caspase-3 antibody (Cell Signaling Technology Inc. Danvers, MA). Anti-GAPDH antibody (Sigma-Aldrich, St Louis, MI) was used as loading control. The bands were quantified by densitometry and the results were normalized with respect to controls run in the same gel.

### [^3^H]-Ryanodine binding

[^3^H]-ryanodine binding was measured in whole homogenates from frozen hearts (0.5 mg protein/mL) following 90 min incubation at 37°C with 10 nM [^3^H]-ryanodine (Perkin Elmer, Boston, MA) in 150 mol/L KCl, 0.5 mmol/L AMPPNP at pCa 5, as previously described [20].

### Infarct size

Infarct size was assessed by the triphenyltetrazolium chloride (TTC, Sigma-Aldrich, St Louis, MI) technique as described [21].

### Statistical analysis

Data are expressed as mean ± S.E.M. Statistical data were analyzed by ANOVA followed by Tukey post test. Differences were considered significant at p<0.05.

## Results

### Dose dependent effects of proteasome inhibitors on hemodynamic parameters and infarct size after ischemia reperfusion

**Fig 1:**
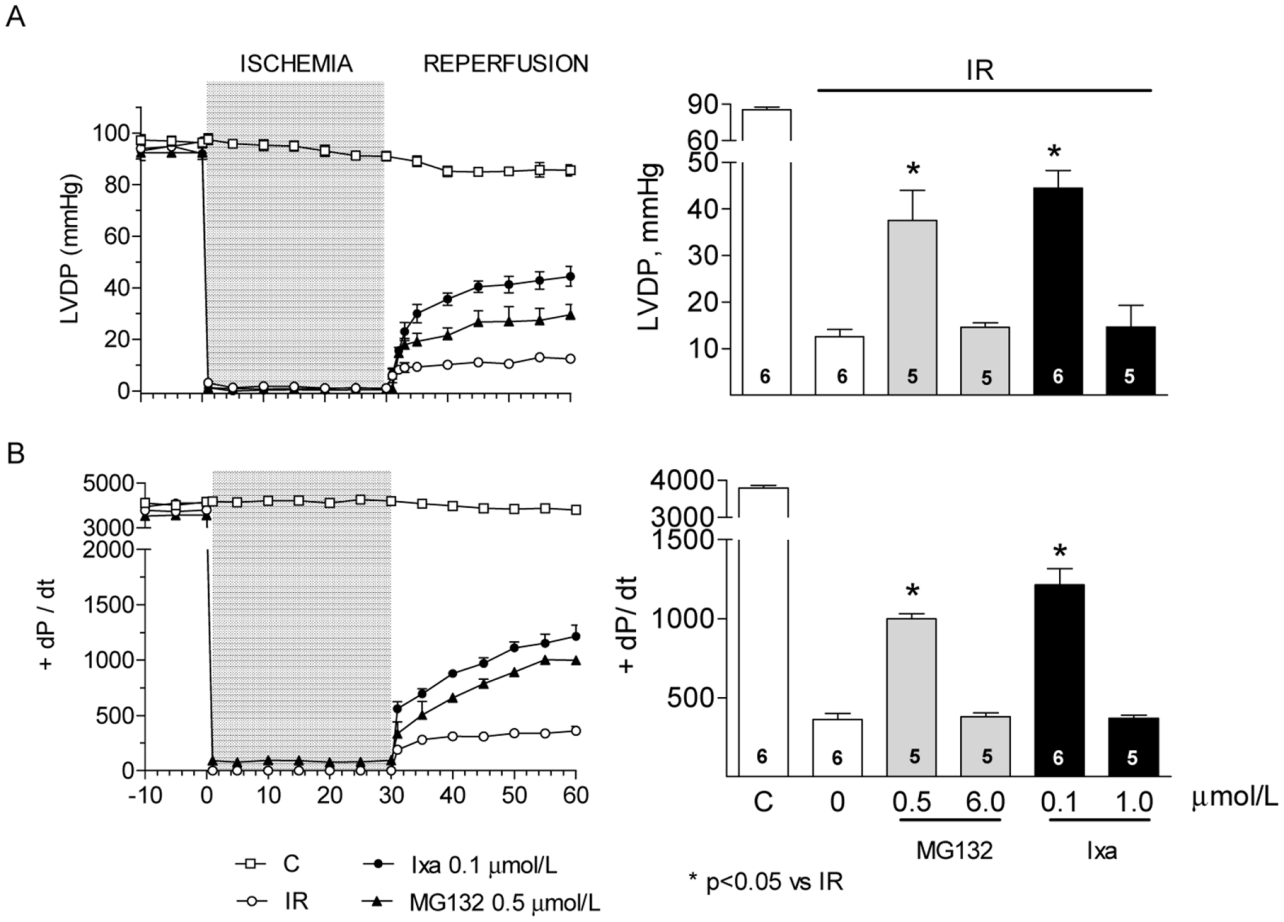
Effect of proteasome inhibition on hemodynamic parameters after ischemia reperfusion. (A) Left ventricular developed pressure (LVDP) and (B) maximal rates of contraction (+ dP/dt) measured in control hearts or after IR with or without the indicated proteasome inhibitor. Bar graphs show the mean ± S.E.M of values measured at 60 minutes of reperfusion of the number of hearts shown in each bar. * p< 0.05 vs IR without inhibitor.

**Fig 2:**
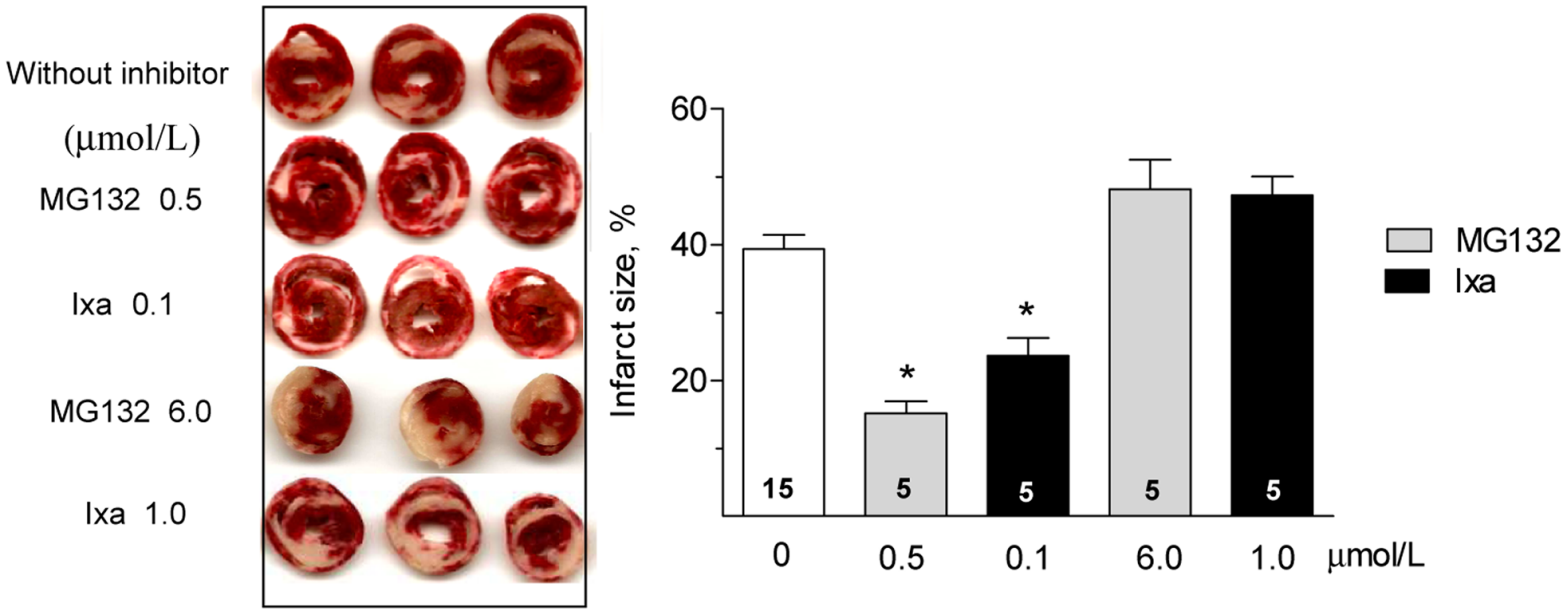
Effect of proteasome inhibition on the infarct size. Representative heart slices stained with TTC after IR (left) with or without the indicated proteasome inhibitor. The bar graph shows the mean ± S.E.M. of the infarct size as % of total heart volume calculated in hearts like those shown at the left. Number of hearts analyzed in each condition is shown in each bar. *p< 0.05 vs IR without inhibitor.

The divergent effects of proteasome inhibitors on the cardiac parameters after IR reported in the literature may arise from different models that employ different types and concentrations of inhibitors. We therefore compared the effect of ixazomib with the widely used classical proteasome inhibitor MG132. In the absence of inhibitors, isolated rat hearts subjected to 30 minutes of non-flow global ischemia at 37°C displayed a severe contractile dysfunction upon reperfusion. After 60 minutes of reperfusion, the left ventricular developed pressure (LVDP, Fig 1A) and the maximum rate of left ventricular pressure rise (+dP/dt, Fig 1B) decreased by 85 and 90%, respectively, relative to the pre ischemic values. Perfusion of hearts with ixazomib for 10 minutes before ischemia produced a biphasic effect: at low concentration (0.1 μmol/L), ixazomib significantly improved LVDP (Fig 1A) and +dP/dt after IR (Fig 1B). At high concentration (1 μmol/L), however, this protective effect was lost and both, LVDP and +dP/dt, were not different to the values found after IR in the absence of inhibitor (Fig 1A and 1B). The same effect, protection at low concentration (0.5 μmol/L) and loss of protection at high concentration (6 μmol/L), was observed when MG132 was administered before ischemia (Fig 1A and 1B). At the concentrations used in this work, neither ixazomib nor MG132, at 0.5 μmol/L, produce significant changes in hemodynamic parameters in control hearts, not subjected to ischemia (S1 Fig). To corroborate the protective effect of a low concentration of proteasome inhibitors, we measured the infarct size after IR. In the absence of inhibitors, the infarct size was 39±8% of the cardiac volume (Fig 2). MG132 and ixazomib significantly reduced the infarct size to15±8% and 24±6% of the heart volume, respectively, when administered at a low concentration, but did not protect at higher concentration (Fig 2). Therefore, ixazomib and MG132 exert a similar dose dependent cardioprotective effect. For this reason we used only ixazomib in most of the following experiments. No measurable infarction was detected in control hearts, perfused with Krebs Henseleit solution with or without proteasome inhibitors.

### Determination of proteasome activity

**Fig 3:**
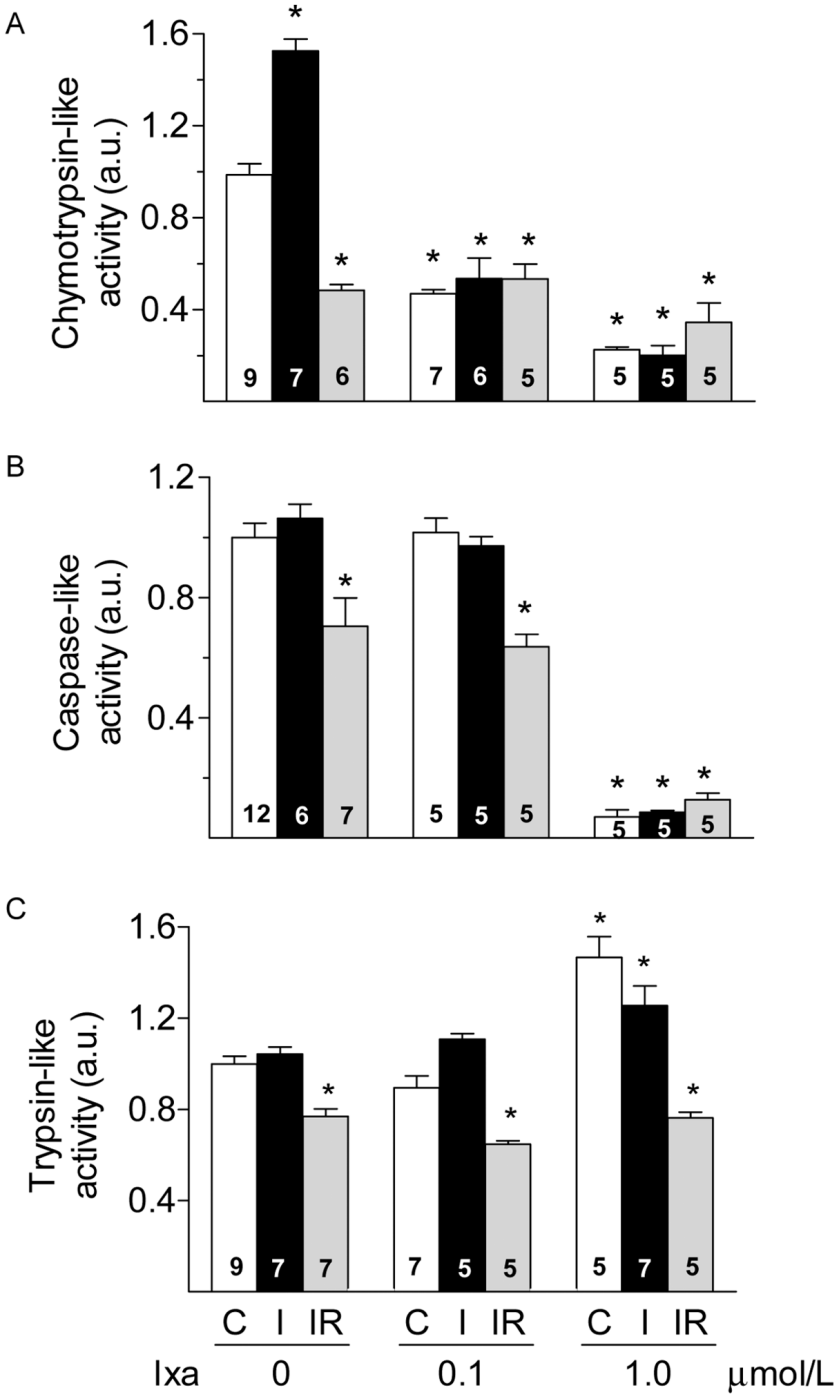
Proteasome activities after I or IR. Chymotrypsin-like (panel A), caspase-like (panel B) and trypsin-like (panel C) activities of the proteasome are shown in controls (C, white bars), after 30 min of ischemia (I, black bars) or after 30 min of ischemia followed by 60 min of reperfusion (IR, grey bars) in hearts perfused with the indicated concentration of ixazomib. Activities were normalized with respect to controls. Bars show the mean ± S.E.M. of different hearts as indicated in each bar. *p< 0.05 vs C without inhibitor.

The results shown above suggest that proteasomes are key players in the damage induced by IR and the biphasic effect of the inhibitors may be produced by different inhibition of proteasome peptidases at the different concentrations of ixazomib or MG132 used. Reports in the literature consistently show decreased proteasome activity after reperfusion (reviewed in [22]), but there is less information on the effect of ischemia on proteasome activity. Therefore we determined the effect of ixazomib on the activities of the 20S proteasome at the end of ischemia or at the end of the reperfusion period. We made the novel observation that after 30 minutes of ischemia in the absence of inhibitor, and before reperfusion, the chymotrypsin like (CT–like) activity was significantly increased by 50% compared to the value observed in control hearts (Fig 3A). Ischemia did not produce changes in the caspase-like (C-like) or the trypsin-like (T-like) activities of the proteasome (Fig 3B and 3C). In contrast, after reperfusion all three main activities of the proteasome were significantly inhibited. On average, we observed reductions of 53%, 30% and 23% for CT-like, C-like and T-like activities respectively (Fig 3A–3C). In hearts perfused with ixazomib, 0.1 μmol/L, the CT-like activity was reduced approximately by half of the basal value both, in control and after 30 min of ischemia. Ixazomib did not caused further reduction of this activity upon reperfusion (Fig 3A). The C-like and the T-like activities of the proteasome were not inhibited by ixazomib 0.1 μmol/L, compared to the same condition without the inhibitor (Fig 3B and 3C). Increasing ixazomib to 1 μmol/L produced a further reduction in CT-like activity to about 25% of the basal value (Fig 3A) and also inhibited the C-like activity to less than 10% of the activity in the absence of inhibitor (Fig 3B). In contrast, perfusion of hearts with ixazomib 1 μmol/L increased the T-like activity of the proteasome in control hearts by 47% (Fig 3C). After 30 min of ischemia, the T-like activity remained significantly elevated but decreased after reperfusion to the value observed in the absence of inhibitor (Fig 3C). In summary, a low concentration of ixazomib (0.1 μmol/L), prevents the increase in CT-like activity induced by ischemia without inhibiting the C-like or T-like activities, while a higher concentration of ixazomib (1 μmol/L) significantly reduces both, the CT-like and C-like activities and increases the T-like activity of the proteasome under basal conditions and this activity remained elevated after 30 min of ischemia. Besides, ixazomib increased the content of ubiquitinated proteins in whole ventricle homogenates of control hearts in a dose dependent manner (S2 Fig), indicating that proteasomes were effectively inhibited by the concentrations used in this work. These results suggest that partial inhibition of CT-like activity during ischemia protects from IR damage, but simultaneous inhibition of CT-like and C-like activities is detrimental. The simultaneous increase in T-like activity may also contribute to the loss of the protective effect of ixazomib.

### Effect of proteasome inhibition on RyR2 protein content

**Fig 4:**
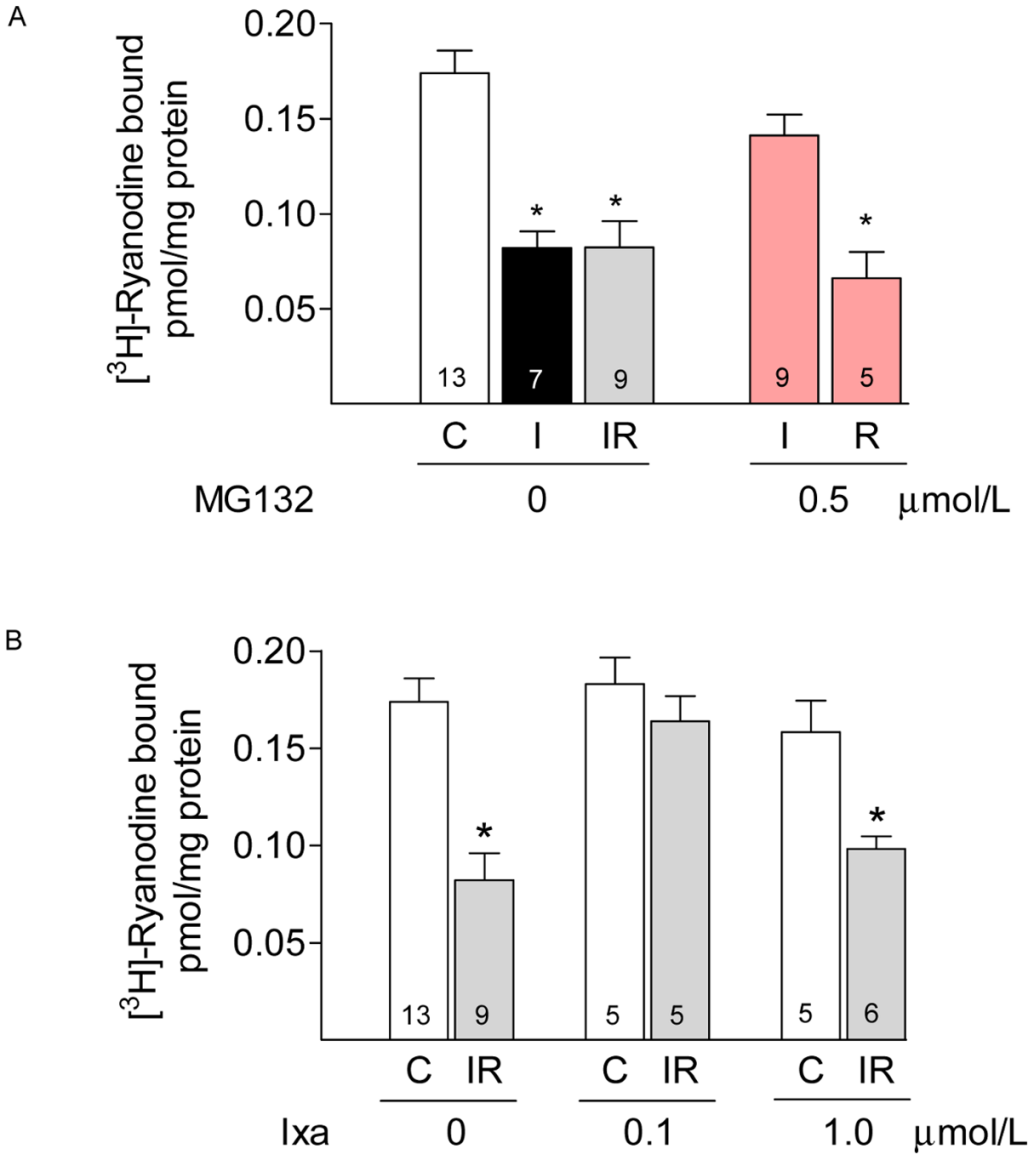
Effect of proteasome inhibitors on [^3^H]-ryanodine binding. Panel A. Specific [^3^H]-ryanodine binding was measured in whole ventricle homogenates obtained in controls (C, white bars), after 30 min of ischemia (I, black bars) or after 30 min of ischemia followed by 60 minutes of reperfusion (IR, grey bars). MG132 was perfused at 0.5 μmol/L before ischemia (red bar, I) or from the very start of reperfusion (red bar, R). Values represent the mean ± S.E.M of different heartsas indicated in each bar *: p< 0.05 vs control or vs I + MG132. Panel B. Specific [^3^H]-ryanodine binding measured in hearts with the indicated concentration of ixazomib (ixa) perfused before ischemia. Values represent the mean ± S.E.M of values obtained in different hearts as indicated in each bar. *: p< 0.05 vs control.

**Fig 5:**
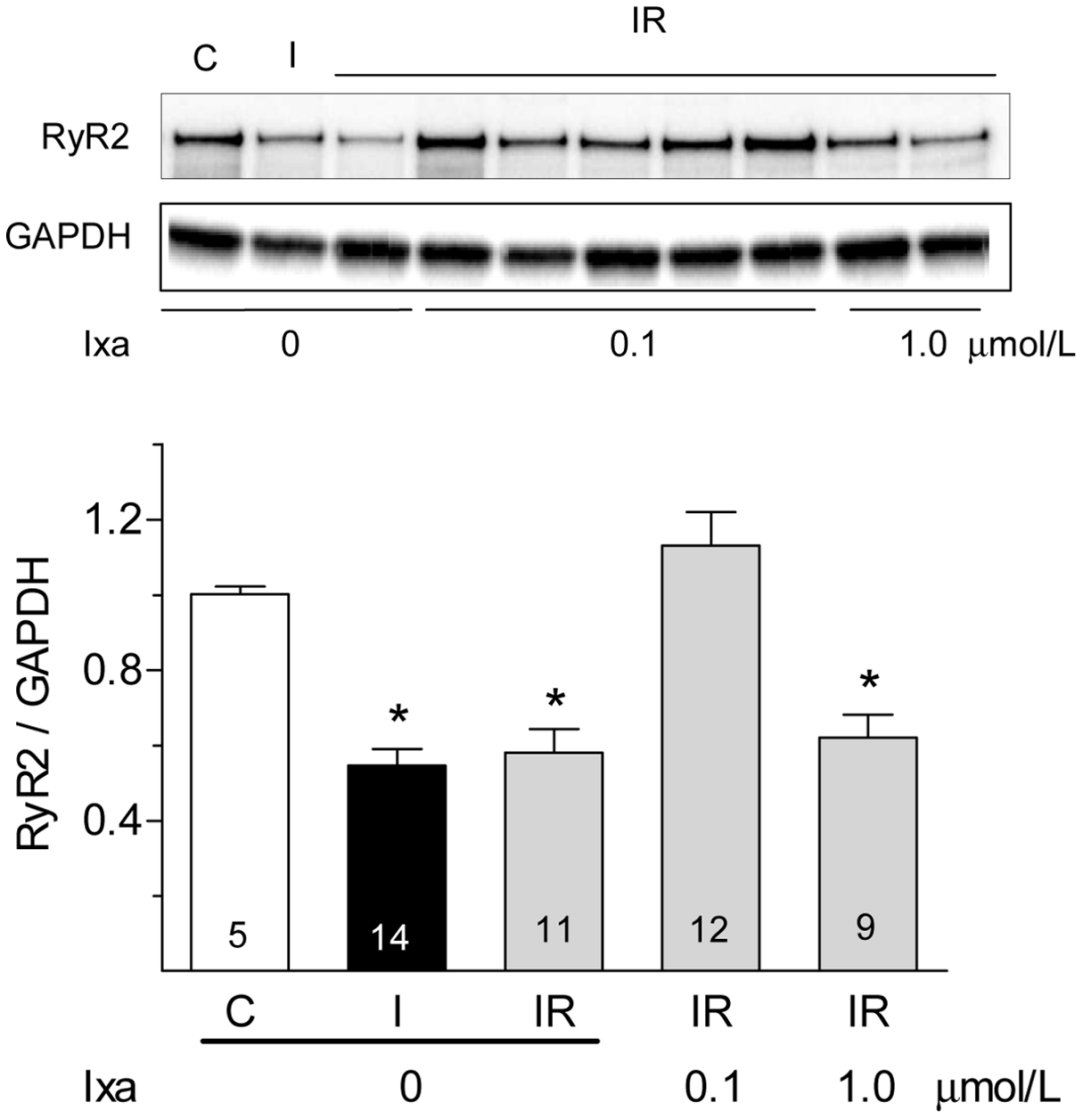
Effect of ixazomib on RyR2 protein content. Representative western blots show RyR2 content in whole ventricle homogenates obtained from controls hearts (C), after ischemia (I), after ischemia-reperfusion (IR) with the indicated concentration of ixazomib (ixa). Graph bar show the mean ± S.E.M of values obtained in different hearts as indicated in each bar. Results were normalized by the content of GAPDH in the same membrane.*: p < 0.05 vs control.

RyR2, the calcium release channel of the sarcoplasmic reticulum is reduced by half after myocardial ischemia in adult hearts [23,24] or after simulated IR in neonatal cardiomyocytes [25]. MG132 preserves the content of RyR2 in neonatal cardiomyocytes, suggesting that proteasomes are involved in the reduction of this protein during ischemia [25]. Therefore, we used RyR2 as a representative protein to corroborate the protective effect of ixazomib during ischemia. We measured RyR2 activity and protein content after ischemia or reperfusion by measuring the binding of [^3^H]-ryanodine and by performing western blots analysis of whole heart ventricles. We found that 30 minutes of global ischemia caused 50% decrease in [^3^H]-ryanodine binding density. No further decrease was observed upon reperfusion (Fig 4A). MG132, 0.5 μM, prevented the decrease in [^3^H]-ryanodine binding when perfused before ischemia but not when added from the very start of reperfusion (Fig 4A, red bars). Ixazomib, which did not modify [^3^H]-ryanodine binding in control hearts, prevented the decrease in [^3^H]-ryanodine binding when perfused before ischemia at 0.1 μmol/L but not at 1 μmol/L (Fig 4B). Since ryanodine binds preferentially to the open conformation of RyR2, its binding depends on the functional state of the channels. To test if the decrease in [^3^H]-ryanodine binding represents a true decrease in RyR2 protein content and does not result from RyR2 modifications that occurred during ischemia and affected the opening of the channel, we also quantified RyR2 content in western blots. As shown in Fig 5, RyR2 protein content decreased by 50% after ischemia and remained at this level after reperfusion; the presence of ixazomib during ischemia prevented the decrease in RyR2 at 0.1 but not at 1 μmol/L. These results strongly suggest that preventing the increase in CT-like activity of the proteasome with ixazomib avoids the loss of RyR2 and other cellular proteins producing the observed beneficial effects.

### Activation of caspase 3 by ixazomib

**Fig 6:**
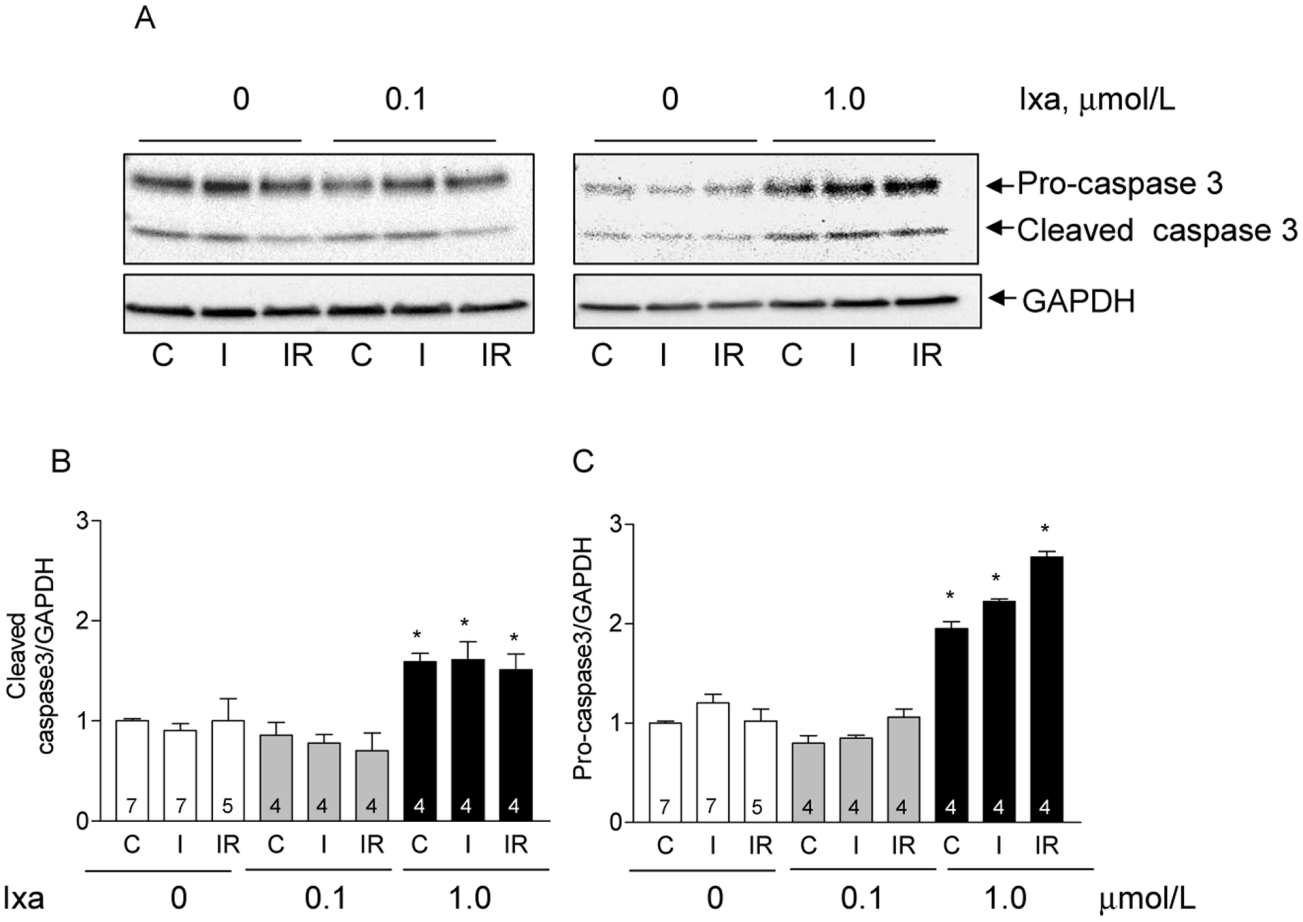
Effect of ixazomib on the activation of caspase3. Representative western blots (A) show cleaved caspase 3 and procaspase 3 content in whole ventricle homogenates obtained from controls hearts (C), after ischemia (I), and after ischemia-reperfusion (IR) with the indicated concentration of ixazomib (ixa). Graph bars show the mean ± S.E.M of cleaved caspase 3 (B) or procaspase 3 (C) obtained in different hearts as indicated in each bar. Results were normalized by the content of GAPDH in the same membrane. *: p < 0.05 vs control without inhibitor.

Inhibition of the proteasome induces apoptosis in different cells [26–28] and the loss of protection by stronger inhibition of the proteasome during myocardial ischemia suggests that a cell death program was activated in this condition. We explored this possibility by measuring caspase-3, an executioner caspase that mediates cellular apoptosis. As shown in Fig 6, a low concentration of ixazomib did not change the cleaved caspase-3 (Fig 6A and 6B) or procaspase-3 content (Fig 6A and 6C) in controls, after ischemia or after ischemia reperfusion; in contrast, ixazomib at 1 μM, increased both, the active caspase-3 content (Fig 6A and 6B) and the total procaspase-3 (Fig 6A and 6C) in hearts subjected to ischemia and in control hearts as well. The increase in active caspase-3 suggests that apoptosis was activated in these hearts with the consequent increase in protein degradation and cell death. The apparent normality of control hearts perfused with high concentration of ixazomib, in spite of the increased active caspase-3, is discussed below.

## Discussion

Our results show that thirty minutes of warm ischemia in isolated rat hearts causes the death of almost 40% of the heart and a poor recovery of hemodynamic parameters upon reperfusion. We found that the CT-like activity of the 20S proteasome increases significantly after 30 min of ischemia, and decreases below control values after reperfusion. The C-like and T-like activities, which did not change during ischemia, decreased as well after reperfusion. These results suggest that the CT-like activity of the proteasome is a privileged target of ischemia-induced injury. The partial inhibition of CT-like activity during ischemia (to 50% of the control value) produced by ixazomib reduced the infarct size by half and produced a significant recovery of hemodynamic parameters, suggesting that during ischemia the 20S proteasome degrades key cellular proteins, such as RyR2, and promotes cell dysfunction and death. Protection was completely lost when the simultaneous inhibition of CT-like and C-like activity was achieved by a higher concentration of ixazomib, which caused a simultaneous activation of T-like activity.

Ixazomib belongs to the second-generation of proteasome inhibitors and it has been recently approved by the FDA for the treatment of multiple myeloma. It inhibits preferentially the CT-like activity of the proteasome over the caspase-like activity (IC50 values of 3.4 and 31 nmol/L, respectively). A higher concentration is required for the inhibition of the T-like activity (IC50 3.5 μmol/L) [29]. The inhibition of the proteasome activities observed in this study is in agreement with these reported inhibitory constants. Further kinetic analysis would be necessary to clarify the molecular basis of the increase in T-like activity observed at the higher concentration of ixazomib. Nevertheless, asimultaneous inhibition of CT-like activity and enhancement of T-like activity is also produced by the inhibitor of the human immunodeficiency virus, type I (HIV-I) protease, ritonavir [30]. This effect would be produced by the binding of ritanovir to a non-catalytic modifier site of the proteasome [30]. A similar mechanism could explain the increase in T-like activity produced by ixazomib.

Previous studies report beneficial [13,14,31], or deleterious [9,10,12] effects of proteasome inhibitors on cardiac IR. The activity of the proteasome was not measured in all these studies and differences in the degree of proteasome inhibition, may explain the discrepancies. As shown here, the same inhibitor can improve or worsen cardiac function after an episode of IR, depending on the degree of inhibition of the different peptidases. Those studies that measure proteasome activity consistently found that ischemia-reperfusion decreases the activity of the proteasome [9,11,12]. We confirmed this inhibition in the present work. In contrast, there is not enough information about the activity of the proteasome after ischemia. Some studies report decreased 26S proteasome activity during ischemia (reviewed in [22]) and only one study reports that the 20S proteasome is also inhibited after ischemia [12]. At difference with that work, we did not use reducing agents in the preparation of heart extracts or in the assay medium. The presence of reducing agents, such as dithiothreitol, may have removed reversible redox modifications like S-glutathionylation that increases the activity of the 20S proteasome [32]. In rat hearts kept in Wisconsin solution at 4°C (cold ischemia), the activity of the 26S proteasome increases more than two fold as ATP content decreases during ischemia [33]. The inhibition of the proteasome at this stage preserves the ultra structural integrity of the hearts and reduces ischemia-reperfusion injury [33] prolonging the viability of the organ for transplant [34]. Cold ischemia did not produce changes in the 20S proteasome [33]. Warm ischemia (37°C), such as that induced here, is a totally different setting because diverse signaling pathways are activated at the beginning of ischemia before the intracellular media changes extensively. In addition, as the ATP concentration decreases, the disociation of the 26S proteasome may increase the abundance of the 20S proteasome, which actively degrades oxidized proteins independently of ATP [35,36].

A detailed elucidation of the molecular mechanisms responsible for the increased proteasome activity produced by ischemia is beyond the scope of the present work. Proteasomes are modulated by a number of post translational modifications [37] and proteomic studies are needed to further clarify this regulation.

Proteasome inhibitors were deliberately designed to produce apoptosis in cancer cells [38,39]. Ixazomib induces apoptosis in multiple myeloma cells through activation of caspases and other proapoptotic proteins such as p53, PUMA and Noxa and proteins involved in the endoplasmic reticulum stress pathway [40]. The increase in the executioner caspase-3 by ixazomib 1 μM, suggests that apoptosis increases when proteasomes are strongly inhibited during ischemia. The lack of visible deleterious effects in control hearts treated with this concentration of ixazomib suggests that apoptosis takes a longer time to complete in normal cells. In fact, proteasome inhibition-induced apoptosis is potentiated by the generation of reactive oxygen species and by increased calcium concentration [27], two conditions that have been amply demonstrated to occur in cardiac ischemia.

In addition, inhibition of the proteasome could have simultaneously activated other cell death pathways such as chaperon mediated autophagy and macroautophagy [41] which may have also contributed to the degradation of RyR2 [42] and to increase the infarct size upon reperfusion [43].

## Supporting Information

S1 Fig Effect of proteasome inhibition on hemodynamic parameters in control hearts. (A) Left ventricular developed pressure (LVDP) and (B) maximal rates of contraction (+ dP/dt) measured in control hearts with the indicated proteasome inhibitor. Inhibitors were perfused at the indicated concentration in oxygenated Krebs Henseleit solution at 37°C and at 10 mL/min during 10 min. Perfusion continued without inhibitor for 90 min, time at which values were measured. Bars show the mean ± S.E.M of 4 different hearts.
(TIF)

S2 Fig Effect of ixazomib on the accumulation of ubiquitinated proteins. Whole ventricle homogenates were analyzed in Western blots for the presence of ubiquitinated proteins after perfusion with the indicated concentration of ixazomib. Total anti-ubiquitin immunoreactivity of each lane was normalized by the content of GAPDH in the same lane. Anti-ubiquitin antibody was obtained from Cell signaling (Danvers, MA). Bars show Mean ± S.E.M values obtained in different hearts as indicated in the bars. *: p < 0.05.
(TIF)
