# Supplementary figures and images for: Activation of Chymotrypsin-Like Activity of the Proteasome during Ischemia Induces Myocardial Dysfunction and Death
Source: PLoS One. 2016 Aug 16;11(8):e0161068. doi: 10.1371/journal.pone.0161068 (PMC4986934; doi:10.1371/journal.pone.0161068)

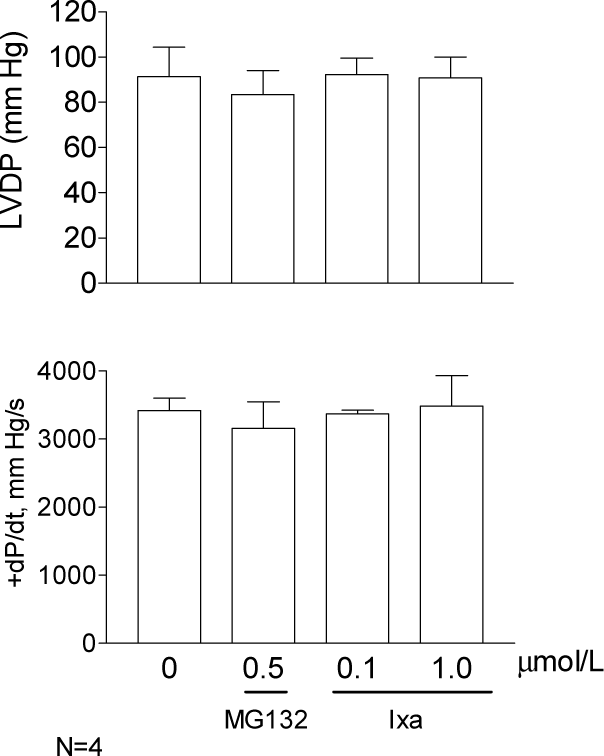

Supplement: S1 Fig — (A) Left ventricular developed pressure (LVDP) and (B) maximal rates of contraction (+ dP/dt) measured in control hearts with the indicated proteasome inhibitor. Inhibitors were perfused at the indicated concentration in oxygenated Krebs Henseleit solution at 37°C and at 10 mL/min during 10 min. Perfusion continued without inhibitor for 90 min, time at which values were measured. Bars show the mean ± S.E.M of 4 different hearts. (TIF) [file pone.0161068.s001.tif]

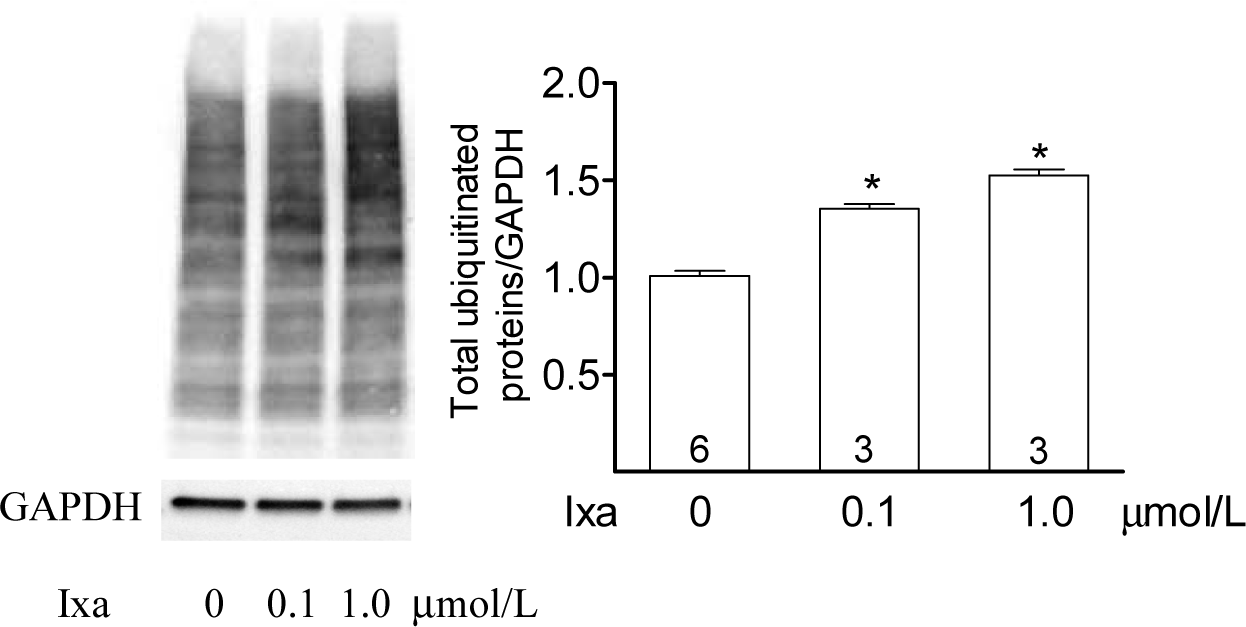

Supplement: S2 Fig — Whole ventricle homogenates were analyzed in Western blots for the presence of ubiquitinated proteins after perfusion with the indicated concentration of ixazomib. Total anti-ubiquitin immunoreactivity of each lane was normalized by the content of GAPDH in the same lane. Anti-ubiquitin antibody was obtained from Cell signaling (Danvers, MA). Bars show Mean ± S.E.M values obtained in different hearts as indicated in the bars. *: p < 0.05. (TIF) [file pone.0161068.s002.tif]
